# Walking With Ears: Altered Auditory Feedback Impacts Gait Step Length in Older Adults

**DOI:** 10.3389/fspor.2020.00038

**Published:** 2020-04-16

**Authors:** Tara Cornwell, Jane Woodward, Mengnan/Mary Wu, Brennan Jackson, Pamela Souza, Jonathan Siegel, Sumitrajit Dhar, Keith E. Gordon

**Affiliations:** ^1^Northwestern University, Biomedical Engineering, Evanston, IL, United States; ^2^Northwestern University, Physical Therapy and Human Movement Sciences, Chicago, IL, United States; ^3^Shirley Ryan AbilityLab, Chicago, IL, United States; ^4^Northwestern University, Communication Sciences and Disorders, Evanston, IL, United States; ^5^Edward Hines Jr. VA Hospital, Research Service, Hines, IL, United States

**Keywords:** locomotion, gait, balance, sound, hearing

## Abstract

Auditory feedback may provide the nervous system with valuable temporal (e. g., footstep sounds) and spatial (e.g., external reference sounds) information that can assist in the control of upright walking. As such, hearing loss may directly contribute to declines in mobility among older adults. Our purpose was to examine the impact of auditory feedback on the control of walking in older adults. Twenty older adults (65–86 years) with no diagnosed hearing loss walked on a treadmill for three sound conditions: Baseline, Ear Plugs, and White Noise. We hypothesized that in response to reduced temporal auditory feedback during the Ear Plugs and White Noise conditions, participants would adapt shorter and faster steps that are traditionally believed to increase mechanical stability. This hypothesis was not supported. Interestingly, we observed increases in step length (*p* = 0.047) and step time (*p* = 0.026) during the Ear Plugs condition vs. Baseline. Taking longer steps during the Ear Plugs condition may have increased ground reaction forces, thus allowing participants to sense footsteps via an occlusion effect. As a follow-up, we performed a Pearson's correlation relating the step length increase during the Ear Plugs condition to participants' scores on a clinical walking balance test, the Functional Gait Assessment. We found a moderate negative relationship (rho = −0.44, p = 0.055), indicating that participants with worse balance made the greatest increases in step length during the Ear Plugs condition. This trend suggests that participants may have actively sought auditory feedback with longer steps, sacrificing a more mechanically stable stepping pattern. We also hypothesized that reduced spatial localization feedback during the Ear Plugs and White Noise conditions would decrease control of center of mass (COM) dynamics, resulting in an increase in lateral COM excursion, lateral margin of stability, and maximum Lyapunov exponent. However, we found no main effects of auditory feedback on these metrics (*p* = 0.580, *p* = 0.896, and *p* = 0.056, respectively). Overall, these results suggest that during a steady-state walking task, healthy older adults can maintain walking control without auditory feedback. However, increases in step length observed during the Ear Plugs condition suggest that temporal auditory cues provide locomotor feedback that becomes increasingly valuable as balance deteriorates with age.

## Introduction

**Figure 1:**
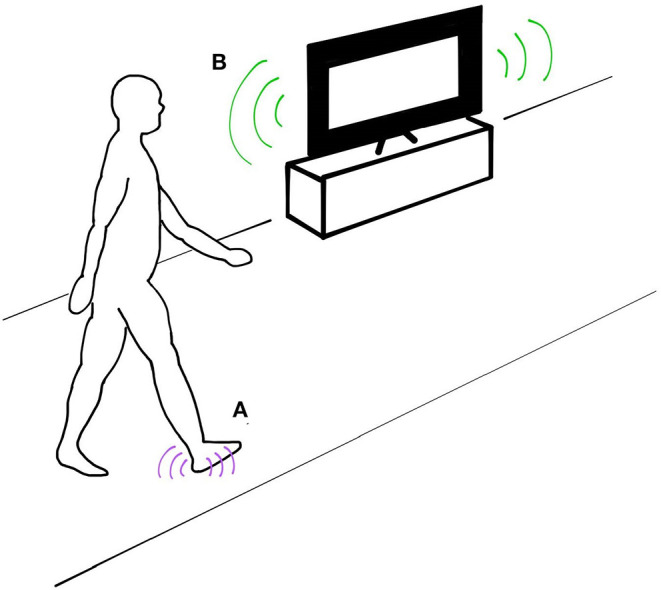
Auditory feedback during walking can provide sensory cues that aid in the control of walking balance. **(A)** Temporal cues such as footsteps provide information about the timing of importance events occurring within the gait cycle. **(B)** External sounds can provide spatial references about an individual's position and velocity.

To maintain and control upright walking, the human nervous system continuously processes and responds to multiple streams of sensory feedback. Although research has focused largely on visual (Warren et al., 2001; McAndrew et al., 2011), vestibular (Fitzpatrick et al., 1999; Bent et al., 2000), and somatosensory feedback (Dietz and Duysens, 2000; Sinkjaer et al., 2000), growing evidence suggests that auditory feedback may also play an important role for controlling gait (Menzer et al., 2010; Baram et al., 2016; Camponogara et al., 2016; Shayman and Earhart, 2017; Weaver et al., 2017) (Figure 1). Sounds produced during walking may provide temporal cues indicating the timing of important events in the gait cycle. For example, footsteps, generated every time the feet contact the ground, mark the critical transition from leg swing to stance. When footstep sounds are artificially delayed, people modulate their walking speed, suggesting that this form of auditory feedback may be important for controlling step frequency (Menzer et al., 2010). Externally-generated environmental sounds may also provide spatial landmarks that can serve as a reference for controlling aspects of walking such as body posture and orientation (Karim et al., 2018). Although the evidence during walking is limited, several studies have found that auditory cues can improve standing postural balance (Easton et al., 1998; Kanegaonkar and Amin, 2012; Rumalla et al., 2015; Horowitz et al., 2019). In addition, the reflection of self-generated sounds associated with walking (i.e., echolocation) could provide information about an individual's absolute position and velocity (Stoffregen and Pittenger, 1995). Given that people's ability to predict location via echolocation improves with movement (Rosenblum et al., 2000), this form of auditory feedback may take on a heightened role during walking in comparison to standing postural tasks when whole-body movements are minimal. Collectively, auditory feedback may be valuable for controlling both temporal and spatial aspects of walking.

Hearing loss may deprive the nervous system of valuable sensory information for controlling walking. Indeed, hearing loss has been associated with poor mobility, including slower walking speeds, problems walking longer distances, and self-reported walking difficulties (Viljanen et al., 2009a; Li et al., 2013). Additionally, cohort studies have identified a positive relationship between hearing loss and the probability of falling (Viljanen et al., 2009b; Lin and Ferrucci, 2012; Girard et al., 2014) with even mild hearing loss (>25 dB) associated with a three-fold increase in fall rate (Lin and Ferrucci, 2012). The relationship between hearing loss and falls appears to hold even when controlling for age (Lin and Ferrucci, 2012), vestibular function (Lin and Ferrucci, 2012), and genetics (Viljanen et al., 2009b). Although the mechanisms have not been fully evaluated, this prior research suggests that loss of hearing is associated with declines in walking and balance. Because the research supporting this relationship is indirect (Viljanen et al., 2009b; Lin and Ferrucci, 2012; Girard et al., 2014), we do not currently know if hearing loss directly causes balance impairments that contribute to falls.

The potential that hearing loss may negatively impact walking balance has important implications for older adults. Among adults over 65 years old, falls are the leading cause of fatal and non-fatal injuries (Houry et al., 2016). The probability of falling increases linearly as the number of risk factors (e.g., muscle weakness or poor vision) increases (Tinetti et al., 1986). Thus, identifying preventable/modifiable risk factors is an essential component of comprehensive fall prevention programs. Despite evidence linking hearing loss to falls (Viljanen et al., 2009b; Lin and Ferrucci, 2012; Girard et al., 2014), hearing is rarely included in fall risk screening and intervention programs (Stevens, 2013). Considering that some forms of hearing loss are addressable by hearing aids or cochlear implants, there is a need to better understand if, and how, hearing loss impacts walking balance in older adult populations.

If auditory feedback is used to control walking, then observable gait modifications may occur in older adults following hearing loss. First, hearing loss may result in more variable step times. Proprioceptive feedback during walking can provide a clear sensory cue indicating when the foot contracts the ground. When the timing of this proprioceptive feedback is modulated, people will modulate the timing of the transitions between stance and swing phases of gait (Pang and Yang, 2000; Gordon et al., 2009). Given that proprioception declines with aging (Shaffer and Harrison, 2007), supplemental sensory feedback such as the sound of footsteps may improve older adults' ability to detect ground contact. If auditory feedback enhances the ability to detect ground contract, then the loss of hearing may result in more variable step timing. Second, older adults may adapt shorter and faster steps when auditory feedback is reduced. These stepping modifications are believed to improve walking balance by reducing center of mass (COM) peak velocities and increasing the opportunity to make corrective steps (Reimann et al., 2018), and are often observed when people walk in challenging and uncertain environments (McAndrew et al., 2010; Wu et al., 2017). If hearing loss reduces the ability of the nervous system to detect ground contact, thus increasing movement uncertainty, then adapting shorter, faster steps could be a simple compensatory strategy to maintain balance. Finally, hearing loss may particularly impact control of lateral COM motion while walking. During normal gait, the COM naturally oscillates from side-to-side as body weight is shifted over the supporting limb (Saunders et al., 1953). To maintain desired forward walking trajectories, research suggests that control of this frontal-plane motion requires greater involvement of the nervous system than control of sagittal-plane motions, which benefit considerably from the human body's passive dynamics (MacKinnon and Winter, 1993; Bauby and Kuo, 2000; O'Connor and Kuo, 2009; McAndrew et al., 2010). Based on this previous research, it is likely that loss of sensory feedback that provides spatial information will have a greater impact on control of COM motion in the frontal plane than in the sagittal plane. Thus, if older adults use auditory feedback to aid in controlling spatial aspects of walking, then hearing loss might result in lateral COM excursions that are larger, faster, and less stable.

The purpose of this study was to examine the impact of auditory feedback on the control of temporal and spatial aspects of walking balance in older adults. Older adults without diagnosed hearing loss were recruited for this within-subjects design. We selected a within-subjects design to minimize the potential effects of confounding factors. Participants walked on a treadmill under conditions in which auditory feedback was reduced by either a combination of ear plugs and over-ear earmuffs or playing white noise (75 dB) through insertable eartips. We hypothesized that compared to baseline walking, a reduction in auditory feedback would affect control of temporal aspects of gait. Specifically, we predicted that step time would be more variable under conditions of reduced auditory feedback and that individuals would adapt shorter, faster steps. We also hypothesized that reductions in auditory feedback would affect spatial control of COM motions during walking. Specifically, we predicted that under conditions of reduced auditory feedback, individuals would increase lateral COM excursion and peak lateral speed per stride and decrease local orbital stability.

## Materials and Methods

### Participants

Twenty-two older adults who did not have hearing aids, cochlear implants, or a known diagnosis of hearing loss were recruited to participate in the study. Additional inclusion criteria included: 50–90 years of age, normal or corrected vision, and ability to walk continuously for 10 min without undue fatigue or health risk. Exclusion criteria included: musculoskeletal and/or vestibular pathologies that would affect balance and/or stability, current use of medication that might affect proprioception and/or balance, and cognitive deficits that preclude understanding of the instructions required to conduct the test. The Northwestern University Institutional Review Board approved the study protocol and all participants provided written informed consent.

### Experimental Setup

We observed participants' gait during a series of walking trials in which auditory feedback was manipulated. Participants walked on a large treadmill, belt size 2.6 m × 1.4 m (Tuff Tread, Willis, TX). For safety, participants wore a trunk harness attached to a passive overhead safety support that allowed free fore-aft motion and was adjusted so it did not restrict small lateral motions or provide bodyweight support (Aretech, Ashburn, VA). Participants did not have access to handrails or other forms of external support during any of the walking trials.

To quantify treadmill walking kinematics, we used a 12-camera motion capture system (Qualisys, Gothenburg, Sweden) to record 3D coordinates of reflective markers placed on the pelvis and feet. Each participant wore a total of 13 reflective markers that we placed bilaterally on the greater trochanters, calcanei, lateral malleoli, 2nd metatarsals, and 5th metatarsals with three additional tracking markers placed on the pelvis. The motion capture system sampled kinematic data at 200 Hz.

### Protocol

We performed three clinical assessments before treadmill testing to evaluate participants' hearing, vision, and walking balance. First, to measure hearing sensitivity, we used custom software operable through a standard web browser. Calibrated pure tones at the frequencies of 0.25, 0.5, 1, 2, and 3 kHz were delivered through ER-2 (Etymotic Research Inc., Elk Grove Village, IL) audiometric earphones. Testing was performed in a clinical laboratory space that was quiet but not soundproof. Prior to performing the hearing sensitivity test, we inspected each participant's ear canals with an otoscope to ensure the ear canal was free of excessive cerumen. Participants were excluded if the ear canal had excessive cerumen buildup. Next, we assessed visual acuity with visual correction using a Snellen eye chart. Finally, to assess walking balance, a licensed physical therapist administered the Functional Gait Assessment (FGA). The FGA is a 10-item test that scores participants' performance on a variety of walking tasks that challenge balance and stability. The FGA has been shown to have excellent reliability and construct validity in older adult populations (Walker et al., 2007; Wrisley and Kumar, 2010; Leddy et al., 2011).

Next, we determined participants' preferred treadmill walking speeds using a staircase method of increasing and decreasing the treadmill speed until a desired speed was verbally confirmed by the participant. Participants walked for two additional minutes at the preferred speed to familiarize them with the setup. All future treadmill walking trials were performed at participants' preferred speed.

Participants then performed 6 or 10 individual 3-min treadmill walking trials. Participants were given rest breaks between trials as needed. During the walking trials, we manipulated two variables: auditory feedback and arm swing. The order of all walking conditions was randomized using a custom MATLAB (Mathworks, Natick, MA) script. There were three auditory feedback conditions: **Baseline**, **Ear Plugs**, and **White Noise**. During the Baseline condition, participants were exposed to the normal ambient sounds of the laboratory. No external devices that could alter hearing were worn during the Baseline condition. During the Ear Plugs condition, participants wore a combination of 3M^TM^ E-A-R^TM^ Classic^TM^ Earplugs with a noise reduction rating (NRR) of 29 dB (3M, Maplewood, MN) and 3M^TM^ PELTOR^TM^ Earmuffs (3M, Maplewood, MN) with an NRR of 27 dB. During the White Noise condition, we created a continuous 75 dB SPL white noise auditory stimuli using Max 7 software (Cycle'74, Walnut, CA). Participants listened to the white noise via ER-2 Insert Earphones with 13 mm Eartips (Etymotic Research, Inc., Elk Grove Village, IL). The wires from the earphones worn in the White Noise condition were fixed to the participants' back to restrict their movement and then manually supported by a researcher positioned behind the study participant. The researcher maintained some slack in the earphone wire to reduce the likelihood that the wire position provided haptic feedback. We used these two methods of reducing auditory feedback based on previous research suggesting that headphones or ear plugs could create an occlusion effect that allows perception of footsteps through bone conduction hearing (Durgin and Pelah, 1999; Hamacher et al., 2018). We thus included a White Noise condition as an alternative method to reduce auditory feedback that would mask bone conduction hearing (Studebaker, 1962). A subset of participants also walked during two additional auditory conditions that included a tone panning between the left and right insert earphones at varying frequencies. These panning trials were not included in the current analysis.

Participants repeated each auditory condition during two arm swing conditions: **Arms Free** and **Arms Crossed**. During the Arms Free condition, participants were instructed to let their arms swing naturally. During the Arms Crossed condition, participants folded their arms across their chest. We included these variations in arm swing due to concerns that the effect of changing auditory feedback on gait might be difficult to detect in a homogenous treadmill environment that provides minimal external challenges to balance. Some research has suggested that arm swing may assist in stabilizing gait (Ortega et al., 2008; Delabastita et al., 2016). We theorized that restricting arm swing in older adults may increase the challenge of controlling walking and in turn increase the role of auditory feedback (i.e., reliance on auditory feedback to control COM motion will be greater when the passively stabilizing effect of arm swing is removed). Thus, we included the Arms Crossed condition in an effort to augment the potential impact of the different auditory conditions on gait. However, it should be noted that other research has found conflicting results and suggests that restricting arm swing may not have significant effects on gait stability (Bruijn et al., 2010).

### Data Analysis

We processed kinematic marker data using both Visual3D (C-Motion, Germantown, MD) and custom MATLAB scripts. The data were gap-filled (third-order polynomial with a maximum gap of 10 frames) and low-pass filtered (fourth-order Butterworth with a cut-off frequency of 6 Hz). The time of heel strike (HS) and toe-off (TO) events were then identified for each step based on the vertical position of the calcaneus marker, and the fore-aft position of the 2nd metatarsal marker, respectively (Wu et al., 2017, 2019). We visually inspected all gait events to ensure accuracy. COM lateral position was estimated as the midpoint of the lateral positions of the two greater trochanter markers. We then calculated lateral COM velocity as the derivative of COM position.

To assess changes in control of temporal aspects of gait, we calculated means and variabilities of individuals' step times and step lengths for each walking condition. To assess changes in control of spatial aspects of gait, we examined local stability of lateral COM velocity and calculated mean values of the minimum lateral margin of stability (MOS) per step, peak lateral COM speed per stride, lateral COM excursion per stride, and step width per step for each condition. We focused our analysis on variables related to controlling the lateral movements of the COM based on previous research suggesting that walking requires active control to maintain stability in the frontal plane (MacKinnon and Winter, 1993; Bauby and Kuo, 2000).

We calculated step time as the time between the HS of one foot and the following HS of the contralateral foot. Step length was calculated as the fore-aft distance between the calcanei markers at HS. Step width was calculated as the medio-lateral distance between the lateral malleoli markers at mid-stance.

For our metric of gait stability, we calculated local stability of lateral COM velocity using the maximum Lyapunov exponent. We chose lateral COM velocity because velocity, in contrast to position data, is not affected by non-stationarities. The Lyapunov exponent quantifies the average logarithmic rate of divergence of a system after a small perturbation (Rosenstein et al., 1993; Dingwell and Cusumano, 2000). The short-term local divergence exponent (λs) has demonstrated theoretical and predictive validity in simulation and empirical studies of walking (Bruijn et al., 2013). We calculated λs from the last 236 steps per trial. The number of steps analyzed was selected to maximize a consistent number of steps across all participants and trials. For construction of a state space, we used a time delay of 10 samples and an embedding dimension of 5. Within this state space, nearest neighbors were identified, and their divergence tracked. From these distances a log (divergence) curve was calculated, and the local divergence exponent was calculated as the slope of this curve between 0 and 0.5 strides (Stenum et al., 2014).

We calculated the minimum lateral MOS per step as the distance between the lateral extrapolated center of mass position (XCOM), a velocity-weighted lateral COM position, and the base of support (Hof et al., 2005) defined by the lateral position of the 5th metatarsal marker during stance phase. The methods used to calculate minimum lateral MOS have been previously described in detail (Wu et al., 2015). This simple inverted pendulum model of walking provides insight into how individuals control frontal-plane motions during walking. When the XCOM is within the BOS, the inverted pendulum will passively self-stabilize. When the XCOM position exceeds the BOS, corrective actions will be required to remain upright. In addition, the impulse required to move the XCOM beyond the BOS will be proportional to the magnitude of the MOS (Hof et al., 2005).

### Statistical Analysis

To investigate how auditory feedback affects gait, we performed separate two-way repeated measures ANOVAs to test for differences in step time, step length, λs, minimum lateral MOS, COM excursion, peak lateral COM speed, and step width between walking trials. The two independent variables were *auditory feedback*, which had three levels—Baseline, Ear Plugs, and White Noise—and *arm swing*, which had two levels—Arms Free and Arms Crossed. We checked each variable for sphericity using Mauchly's sphericity test. If sphericity was violated, we used the Greenhouse-Geisser (GG) correction and *p*-value to test the main effect of auditory feedback and interaction effect of arm swing. When a significant main effect of auditory feedback was found, Bonferroni-corrected pairwise comparisons were performed to look for differences between the Baseline condition and the other two conditions. When a significant main effect of arm swing was found, a *t*-test was performed to compare Arms Free and Arms Crossed. When a significant interaction of auditory feedback and arm swing was found, simple effects analysis was conducted (i.e., a Bonferroni-corrected *t*-test compared Baseline vs. the other two auditory feedback conditions within each arm swing condition). For any measures with distributions that could not be determined as normal, a Friedman's two-way test substituted for the repeated measures ANOVA. The resultant chi-square and *p*-value were recorded. Significance was set at the *p* < 0.05 level for the repeated measures ANOVAs, pairwise comparisons, and *t*-tests.

## Results

### Participants

Of the 22 enrolled participants, 20 completed the study. Two participants were excluded due to significant osteoarthritis with apparent gait impairments (*n* = 1) and excessive cerumen in the ear canal making it unsafe to perform audiometry (*n* = 1). Participants completing the study were 76.9 ± 6.4 years of age (mean ± standard deviation), 7 male/13 female, and had FGA scores of 25.7 ± 2.5. Average performance on the FGA exceeded the established cutoff score of 22/30 for predicting unexplained falls in community-dwelling older adults (Wrisley and Kumar, 2010). Hearing thresholds were recorded at 0.25, 0.5, 1, 2, and 3 kHz. Results of the audiometry testing found participants had hearing thresholds with an average score of 44.9, 39.4, 28.1, 33.8, and 37.4 dB SPL at 0.25, 0.5, 1, 2, and 3 kHz, respectively. Thresholds at 0.25 and 0.5 kHz may have been adulterated due to environmental noise interference. The average hearing thresholds between 1 and 3 kHz were consistent with a gently sloping mild hearing loss. The participants with the worst hearing thresholds exhibited moderate hearing loss. Of the participants completing the full study, all were able to successfully perform the walking tasks without assistance or incidence of falls. Full demographic data can be found in the data repository (https://digitalhub.northwestern.edu/collections/a0deab15-7c16-4c52-86f8-80c96a2fb888).

### Temporal Metrics

**Figure 2:**
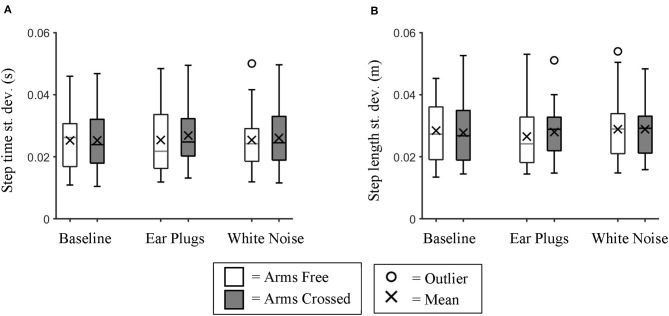
Variability of Step time and Step Length. Box plot data for all participants showing the changes in **(A)** step time variability and **(B)** step length variability between auditory feedback and arm swing conditions. We found no significant differences in variability for either variable between conditions.

We found no significant differences in step time variability (repeated measures ANOVA; *p* = 0.688) (Figure 2A) or step length variability (Chi-squared; *p* = 0.367) (Figure 2B) between auditory feedback conditions.

**Figure 3:**
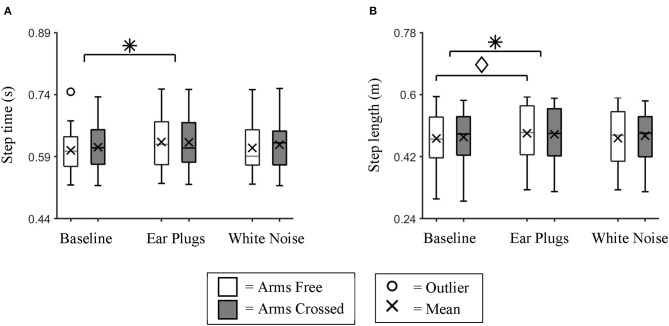
Mean Step time and Step Length. Box plot data for all participants showing the changes in **(A)** mean step time and **(B)** mean step length between auditory feedback and arm swing conditions. We found individuals took significantly slower and longer steps during the Ear Plugs condition than during Baseline. * indicates a significant main effect (*p* < 0.05) between auditory feedback conditions. ♢ indicates a significant simple effect (*p* < 0.05) between auditory feedback conditions during Arms Free walking trials.

Mean step time and mean step length were both affected by auditory feedback. We found a significant main effect of auditory feedback (repeated measures ANOVA; *p* = 0.032) on step time (Figure 3A). *Post hoc* testing identified that step time was significantly longer (Bonferroni-corrected *t*-test; *p* = 0.026) for the Ear Plugs condition than the Baseline condition.

We also found a significant main effect of auditory feedback on step length (repeated measures ANOVA; *p* = 0.037). *Post hoc* testing identified that participants took significantly longer steps during the Ear Plugs condition than the Baseline condition (Bonferroni-corrected *t*-test; *p* = 0.047) (Figure 3B). There was also a significant interaction effect between auditory feedback and arm swing for step length (repeated measures ANOVA; *p* = 0.036). *Post hoc* testing identified that within the Arms Free condition, participants took significantly longer steps during the Ear Plugs condition than the Baseline condition (Bonferroni-corrected *t*-test; *p* = 0.031).

**Figure 4:**
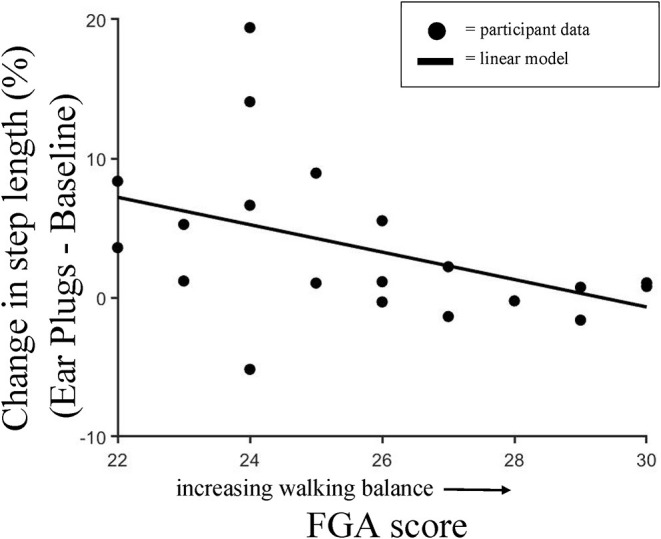
Change in Step Length vs. FGA score. Pearson's correlation showing individual changes in step length observed between the Baseline and Ear Plugs condition and walking balance as assessed by the Functional Gait Assessment score. We found a moderate negative relationship that approached significance (rho = -0.44; *p* = 0.055), indicating that individuals with the poorest balance increased step length the most during the Ear Plugs condition.

To further examine the changes in step length observed between the Baseline and Ear Plugs condition, we calculated a Pearson's correlation between the changes in step length (Ear Plugs—Baseline condition) and individuals' walking balance as assessed by the FGA (Figure 4). We found a moderate negative relationship that approached significance (rho = −0.44; *p* = 0.055), indicating that individuals with the poorest balance increased step length the most during the Ear Plugs condition.

### Spatial Metrics

**Figure 5:**
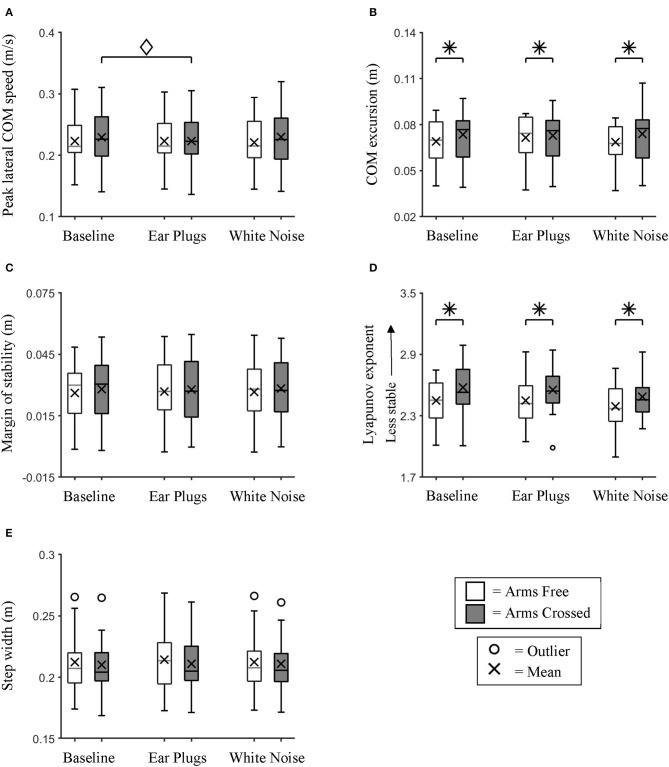
Lateral Center of Mass Dynamics. Box plot data for all participants showing the changes in **(A)** Peak lateral COM speed, **(B)** lateral COM excursion, **(C)** minimum lateral margin of stability, **(D)** λs, and **(E)** step width between auditory feedback and arm swing conditions. We found no significant main effects of auditory feedback on control of lateral COM dynamics. * indicates a significant main effect (*p* < 0.05) between arm swing conditions. ♢ indicates a significant simple effect (*p* < 0.05) between auditory feedback conditions during the Arms Crossed walking trials.

We found no significant main effect of auditory feedback on any of the five spatial metrics analyzed; λs (repeated measures ANOVA; GG *p* = 0.056), minimum lateral MOS, lateral COM excursion, peak lateral COM speed (repeated measures ANOVAs; all *p* > 0.05), and step width (Chi-Squared; *p* = 0.535) (Figure 5).

We found a significant main effect of arm swing on λs (repeated measures ANOVA; *p* < 0.0005) and COM excursion (repeated measures ANOVA; *p* = 0.016) (Figure 5). *Post hoc* testing identified that participants were significantly less stable (*t*-test; *p* < 0.0005) and had larger COM excursions (*t*-test; *p* = 0.016) during the Arms Crossed than the Arms Free conditions.

We found significant interaction effects between auditory feedback and arm swing for COM excursion (repeated measures ANOVA; *p* = 0.005) and COM speed (repeated measures ANOVA; *p* = 0.024). However, *post hoc* testing found no significant differences in COM excursion between auditory conditions within either of the arm swing conditions (Bonferroni-corrected *t*-test; *p* > 0.05). Within the Arms Crossed condition, COM speed was significantly larger during Baseline than the Ear Plugs condition (Bonferroni-corrected *t*-test; *p* = 0.031).

## Discussion

We examined the impact of auditory feedback on control of temporal and spatial aspects of walking in older adults. We hypothesized that older adults would exhibit more variable step timing and adapt shorter, faster steps during treadmill walking when auditory feedback was reduced with either ear plugs or white noise. These hypotheses regarding the temporal aspects of gait were not supported. Step time and length variabilities were not affected by changes in auditory feedback, and contrary to our hypothesis, individuals took longer steps when wearing ear plugs. We also hypothesized that spatial control of lateral COM motions during walking would be negatively affected by reductions in auditory feedback. This hypothesis was also not supported. We observed no significant main effects of auditory feedback on lateral COM motion.

### Temporal Control

Sounds created by our footsteps can provide information about temporal aspects of walking. Previous research has found that people will modify their walking speed when the sounds of their own footsteps are artificially delayed (Menzer et al., 2010) or altered to resemble different terrains (Turchet et al., 2013). In response to clear auditory feedback of one's own footsteps, individuals with cerebral palsy (Baram and Lenger, 2012), multiple sclerosis (Baram and Miller, 2006), and Parkinson's disease (Baram et al., 2016) adapt longer strides and faster walking speeds. Augmenting footstep sounds has also been found to reduce step length variability in individuals with Parkinson's disease (Rodger et al., 2014). Collectively, these previous findings suggest that people use auditory feedback associated with footsteps to control temporal aspects of walking. In the current study, we sought to identify if the removal of auditory feedback during walking would directly affect temporal aspects of walking in older adults.

We hypothesized that reduction of auditory feedback would result in greater step-to-step variations in step timing. This result was not supported by our findings. Neither step time variability nor step length variability changed when auditory feedback was reduced during the Ear Plugs and White Noise conditions. Our findings are similar to the results of Weaver et al. who also failed to identify differences in stride length variability when bilateral hearing aid or cochlear implant users walked with and without their hearing devices (Weaver et al., 2017). These findings suggest that auditory feedback may have a minimal contribution to ongoing control of steady-state walking. Auditory feedback may not have been necessary for controlling step variability because the nervous system receives multiple streams of sensory information, such as proprioceptive feedback received when the foot contacts the ground each step (Pang and Yang, 2000; Gordon et al., 2009), that can provide redundant temporal information to footstep sounds. It is also possible that if individuals in the current experiment had walked in environments with greater challenges to walking (e.g., uneven surfaces) that the role of auditory feedback for controlling temporal aspects of walking would have been greater. A limitation of the current study was that we examined step time variability during treadmill walking, which is known to reduce gait variability (Hollman et al., 2016).

We also hypothesized that when auditory feedback was reduced, individuals would adapt shorter and faster steps. Adapting shorter and faster steps is a common response when people walk in balance-challenging and unpredictable environments (McAndrew et al., 2010; Wu et al., 2017). This strategy can improve an individual's ability to correct movement errors during walking by reducing their COM velocities and increasing the opportunity to make corrective steps (Reimann et al., 2018). We anticipated that individuals would adapt shorter, faster steps based on the assumption that removal of auditory feedback would hinder the nervous system's capacity to accurately sense ongoing walking dynamics, which would result in greater movement uncertainty. Results of this study did not support this hypothesis. To our surprise, compared to Baseline, individuals adapted longer steps during the Ear Plugs condition. Not only did individuals increase step length, but the participants with the poorest balance made the greatest increases in step length when using ear plugs. Of note, reduction of auditory feedback using white noise did not result in significant modifications to step lengths when compared to Baseline. Our finding was similar to the results of Hamacher et al. who observed that older adults wearing noise-canceling headphones actually improved local gait stability in comparison to normal walking (Hamacher et al., 2018). The authors suggested that this unexpected finding could be a result of the noise-canceling headphones creating an occlusion effect that may have improved individuals' ability to perceive their own footsteps or that the headphones reduced cognitive load. We believe that an occlusion effect may best explain our finding that participants adopted longer steps during the Ear Plugs condition but not during the White Noise condition.

It is recognized that when wearing ear plugs or inserts, the sound of an individual's own footsteps often appears amplified. This phenomenon has been explained by an occlusion effect; small vibrations of the ear plugs are transmitted to the basilar membrane, where they are received in a manner similar to air conduction hearing, via either bone conduction or pressure changes in the ear canal (Stenfelt and Goode, 2005; Stenfelt and Reinfeldt, 2007). During walking, the vibratory excitation of the ear plug is a result of the ground reaction force created when the foot contacts the ground. People can modify the profile of their ground reaction force by modifying their speed or walking style (Galbraith and Barton, 1970; Ekimov and Sabatier, 2006), which should change the perception of footsteps via the occlusion effect. Taking longer steps should increase the magnitude of the anterior-posterior component of the ground reaction force (Martin and Marsh, 1992), which we speculate should amplify the occlusion effect experienced during the Ear Plugs condition. Unfortunately, we were not able to quantify the magnitude of the ground reaction forces or the occlusion effect during the walking trials. While we observed this increase in step length during the Ear Plugs condition, we did not during the White Noise condition. One possible explanation is that the white noise masked any occlusion effect (Studebaker, 1962) created by wearing the insert earphones. Therefore, future studies should focus on auditory manipulation methods employing air conduction hearing, such as playing white noise, to reduce the effects of bone conduction hearing. In addition, future studies should also consider implementing methods to directly assess participants' perception of their footsteps in order to evaluate the efficacy of the intended auditory manipulation.

Cognitive load could have played a role in the manipulated auditory feedback conditions. Executive function and attention are known to affect walking performance (Yogev-Seligmann et al., 2008). Among older adults, the addition of cognitive loads can result in decreases in gait stability and balance (LaRoche et al., 2014). Past research suggests that noise can have variable effects on performance of different tasks (Dalton and Behm, 2007). It is possible that the manipulated auditory feedback conditions may have affected attention. However, in the current study, we did not explicitly assess the effects of the auditory conditions on cognitive load, making it difficult to speculate on any potential interactions between auditory feedback conditions, cognitive load and walking performance. The effects of cognitive load should be considered in future studies.

### Spatial Control

Several studies have observed that external environmental sounds can act as a reference for controlling aspects of walking such as body posture and orientation (Karim et al., 2018) and can improve standing postural balance (Easton et al., 1998; Deviterne et al., 2005; Kanegaonkar and Amin, 2012; Gandemer et al., 2014; Rumalla et al., 2015; Horowitz et al., 2019). A considerable body of research has suggested that during walking, control of frontal-plane motion requires greater involvement of the nervous system than sagittal-plane motions (MacKinnon and Winter, 1993; Bauby and Kuo, 2000; O'Connor and Kuo, 2009; McAndrew et al., 2010). As such, we hypothesized that reductions in auditory feedback that may provide spatial information would result in lateral COM excursions that are larger, faster, and less stable. This hypothesis was not supported. We observed no significant differences in lateral COM excursion, peak lateral COM speed, minimum lateral MOS, λs, or step width between auditory feedback conditions. Our findings suggest that continuous auditory feedback may have a limited role in controlling lateral COM dynamics in older adults during a steady-state walking task.

There are several possible reasons why we did not observe changes in control of lateral COM dynamics between auditory conditions. First, although a preferable setup for evaluating steady-state walking, the treadmill environment imposes more restrictions than normal over-ground walking, including an invariable speed, which may have limited our ability to detect gait changes. Additionally, the task may not have sufficiently challenged control of walking balance. To address this issue and introduce a greater challenge to controlling COM dynamics, we added an Arms Crossed condition. While two of our measures, COM excursion and λs, were sensitive enough to detect significant differences between the Arms Crossed and Arms Free conditions, this added challenge was insufficient to detect differences in control between the auditory feedback conditions. It is also possible that other variables, such as body orientation (Karim et al., 2018), may have been more sensitive to the effects of auditory feedback than the measures we selected. We did not measure changes in body orientation because the moving treadmill belt forces individuals to maintain a relatively straight-ahead walking trajectory. Finally, as past research has suggested (Karim et al., 2018), it is possible that the contribution of auditory feedback for controlling walking is increased in situations in which other sensory information is reduced, such as walking in low-light conditions. Future research examining over-ground walking in situations of reduced visibility and auditory feedback would address the limitations of the current study.

### Clinical Implications

Although past research has found a positive relationship between hearing loss and falls (Viljanen et al., 2009b; Lin and Ferrucci, 2012; Girard et al., 2014), the results of the current study do not indicate that reductions in auditory feedback have a significant impact on the ability of healthy older adults to control steady-state walking in a low-complexity environment. It is possible that the role of auditory feedback may take on greater importance for controlling walking in more challenging environments; that is to say, auditory feedback may be more important when responding to discrete challenges, such as detecting a change in walking surface, or in situations when other forms of sensory feedback are reduced. However, our finding that older adults increased step length when walking with ear plugs—which may have facilitated perception of footsteps via an occlusion effect—suggests that older adults may be able to utilize enhanced auditory feedback to improve control of walking balance. This finding aligns with results of several studies that similarly found enhanced auditory feedback of one's own footsteps can improve walking speed, stride length, and gait stability in clinical and older adult populations (Baram and Miller, 2006; Baram and Lenger, 2012; Baram et al., 2016; Hamacher et al., 2018). In addition, the trend that individuals with the poorest walking balance made the greatest increases in step length during the Ear Plugs condition may suggest that the temporal feedback received from footstep sounds is a strategy for controlling balance. If so, gait training performed with enhanced audio feedback of one's own footsteps could be used to improve walking balance of older adults.

### Conclusions

Overall, our results suggest that during a steady-state walking task, healthy older adults are able to maintain walking control without auditory feedback. However, increases in step length observed during the Ear Plugs condition suggest that temporal auditory cues associated with footsteps may provide feedback for controlling walking balance that may be increasingly valuable as balance declines with age.

## Data Availability Statement

The data analyzed for this study can be found at the Northwestern University Feinberg School of Medicine Digital Hub (https://digitalhub.northwestern.edu/collections/a0deab15-7c16-4c52-86f8-80c96a2fb888).

## Ethics Statement

The studies involving human participants were reviewed and approved by Northwestern University Institutional Review Board. The participants provided their written informed consent to participate in this study.

## Author Contributions

MW, PS, JS, SD, and KG conceived of and designed the study. JW recruited participants and performed clinical assessments. TC, JW, MW, and BJ collected and analyzed. All authors were involved in interpretation of data. TC, JW, and KG drafted the manuscript. All authors participated in revising the manuscript.

### Conflict of Interest

The authors declare that the research was conducted in the absence of any commercial or financial relationships that could be construed as a potential conflict of interest.
